# Salting-out re-distillation combined with sensory-directed analysis to recover odor-active compounds for improving the flavor quality of instant Pu-erh tea

**DOI:** 10.1016/j.fochx.2022.100310

**Published:** 2022-04-18

**Authors:** Chao Wang, Juan Li, Ya Zhang, Xuejiao Wu, Zhongrong He, Yin Zhang, Xingmin Zhang, Qin Li, Jianan Huang, Zhonghua Liu

**Affiliations:** a Key Laboratory of Tea Science of Ministry of Education, Hunan Agricultural University, Changsha, Hunan 410128, PR China; b National Research Center of Engineering Technology for Utilization of Functional Ingredients from Botanicals, Hunan Agricultural University, Changsha, Hunan 410128, PR China; c Co-Innovation Center of Education Ministry for Utilization of Botanical Functional Ingredients, Hunan Agricultural University, Changsha, Hunan 410128, PR China; d Yunnan Tasly Deepure Biological Tea Group Co., Ltd, Simao, Yunnan 665000, PR China

**Keywords:** Salting-out re-distillation, Sensory-directed analysis, Odor-active compounds, Instant Pu-erh tea, Flavor quality

## Abstract

The objective of this study was to develop an effective recovery technology of odor-active compounds (OACs) to improve the flavor quality of instant Pu-erh tea (IPT) based on their released behaviors. Salting-out re-distillation (SRD) combined with sensory-directed analysis was developed. The contributing factors, including the soaking time of tea, recovery volume of condensed water of first distillation, amount of sodium chloride, recovery volume of condensed water of SRD, and re-use times of sodium chloride, were studied systematically. Under optimized conditions, 41 OACs were recovered in the first distillation, and the total recovery rate was 83.94%. Forty-one OACs were recovered via SRD, and the total recovery rate reached 72.29%, significantly better than membrane method (33.46%). The IPT prepared by adding OACs that recovered via SRD showed strong aroma attributes intensities and good coordination. This developed method can provide a more effective scheme to improve the flavor quality of IPT

## Highlights

• Salting-out re-distillation was used to recover Pu-erh tea odor-active compounds.
• Various recovery parameters were optimized to achieve an optimal process.
• Effective recovery and concentration odor-active compounds via the developed method..
• The instant Pu-erh tea prepared with the developed method had good flavor quality.

## 1 Introduction

Instant tea industry often generates odorous gas and odorous aqueous effluents released by the processing technology (Wang, He, Zhang, Du, Xiao, & Xu, 2020). Environmental constraints and increased by consumer requirements for the flavor quality of instant tea products have led tea industry to focus on recovering the released odor-active compounds (OACs), achieving cleaner production and obtaining instant tea products with higher flavor quality. Tea aroma is the comprehensive presentation of the interaction of hundreds of different OACs present at very low concentrations (mg/kg level or lower) (Ma et al., 2021), which is an important influencing factor to measure the sensory quality of tea and its deep-processing products (Pang et al., 2019), and directly determines the market value and significantly influence consumers’ perception and acceptance (Deng et al., 2021). During the production of instant tea, failure to effectively recover volatile compounds of tea will seriously affect the flavor quality of final products and cause serious environmental pollution. Therefore, the development of efficient recovery technology of tea aroma compounds is worthy of in-depth study.

Pu-erh tea, a special microbial post-fermented tea, is produced by modern pile-fermentation technology using sun-dried tea leaves of *Camellia sinensis* var. *assamica* (Masters) Kitamura (Theaceae) as the raw material (Chen et al., 2022). The intrinsic quality of tea raw materials has changed dramatically by physicochemical power (damp-heat) and biochemical power (microorganism and enzyme) in the process of fermentation (Li, Feng, Luo, Yao, Zhang, & Zhang, 2018), which has created the unique stale flavor and special health-care effects of Pu-erh tea (Lv, Zhong, Lin, Wang, Tan, & Guo, 2012). However, the composition of volatile compounds of Pu-erh tea are more complex and their contents varies greatly due to its special fermentation process (Pang et al., 2019), and the effective recovery of volatile OACs in the production of instant Pu-erh tea (IPT) is facing greater difficulties.

Several methods, such as membrane technology (Podstawczyk, Mitkowski, Dawiec-Liśniewska, & Witek-Krowiak, 2017), adsorption (Canteli, Carpiné, Scheer, Mafra, & Mafra, 2014), absorption (Brazinha, Alves, Viegas, & Crespo, 2009), and condensation (Wylock, Eloundou Mballa, Heilporn, Debaste, & Fauconnier, 2015), have been used to recover volatile compounds in food industry processing. Among these methods, membrane technology is based on the relative diffusion coefficient and relative solubility in membrane material of each target, and it can effectively separate and recover volatile compounds by selecting appropriate membranes (Yusuf et al., 2020). However, strict membrane pre-treatment, short service life, easy contamination, and high costs limit the application of this technology. Adsorption technology is based on the interaction of volatile compounds and solid matrix to make them adhere to the solid matrix, and this method is generally used to deal with volatile compounds with low concentrations (Karlsson & Trägårdh, 1997). Absorption involves the contact of gas flow with the liquid absorbent and the adsorption of volatile components with liquid matrix, and this method is generally used to recover volatile compounds from gas effluent. However, these two technologies have strong selectivity for the recovery of volatile compounds, and in most cases, only part of volatile compounds can be recovered (Wang, He, Zhang, Du, Xiao, & Xu, 2020). Meanwhile, the final recovery of volatile compounds after adsorption/absorption still needs to be processed with organic solvents. Condensation technology is a commonly used methods for the recovery of volatile compounds from gas mixtures. In food industries, volatile compounds are released with water vapor during processing and converted into aqueous solution via condensation system. However, the recovered volatile compounds are remain highly diluted and require further treatment (Wylock, Eloundou Mballa, Heilporn, Debaste, & Fauconnier, 2015).

Salting-out extraction, an effective separation technique, has been widely used to extract target substances from their aqueous solution with the aid of inorganic salt (Fu et al., 2017). This technique offers many merits, such as environment-friendly, low-costs, energy- and time-saving operation, easy recovery of inorganic salt, easy of scaling up, and continuous operation (Huang et al., 2021). It has been considered as an alternative to the conventional methods for the extraction and separation of bio-based products and natural products (Dai et al., 2021, Jia et al., 2021).

In the present research, the volatile OACs of Pu-erh tea were recovered by using salting-out re-distillation (SRD) concentration. The effects of the soaking time of tea, recovery volume of condensed water of first distillation, amount of sodium chloride, recovery volume of condensed water of re-distillation, and re-use times of sodium chloride were studied systematically. This study aims to effectively recover the OACs released during the production of IPT and obtain high-quality IPT products.

## 2 Materials and methods

### 2.1 Materials

Five representative Pu-erh tea samples (processed in 2014 and 2017) were supplied by Yunnan Tasly Deepure Biological Tea Group Co., Ltd. (Pu’er, Yunnan, China). The samples were evaluated and selected based on “Methodology for Sensory Evaluation of Tea” (Chinese Standards, GB/T 23776–2018) (Table S1). The experiment sample was prepared by mixing all the selected Pu-erh tea with equal weight, and ground by a grinder and passed through 30 mesh before the experiments.

*n*-Hexane (PubChem CID: 8058; ≥98.0%), *n*-butanol (PubChem CID: 263; ≥99.9%), anhydrous sodium sulfate (PubChem CID: 24436; ≥99.0%), (*E*,*Z*)-2,6-nonadienal (PubChem CID: 643731; ≥95%) was purchased Macklin (Shanghai, China). *β*-Linalool (PubChem CID: 6549; 98%), *α*-terpineol (PubChem CID: 17100; >95%), linalool oxide III (PubChem CID: 26396; ≥99%), and *β*-ionone (PubChem CID: 638014; 97%) were purchased from Aladdin (Shanghai, China). 1,2,3-Trimethoxybenzene (PubChem CID: 12462; 98%), geranyl acetone (PubChem CID: 1549778; ≥98%), dihydroactinidiolide (PubChem CID: 27209; 98%), and *n*-alkanes solution (C_8_–C_32_, calculate retention index) were purchased from J&K Chemical (Beijing, China). Citral (PubChem CID: 638011; 98%) and dihydro-*β*-ionone (PubChem CID: 519382; ≥90%) were purchased from Bidepharm (Shanghai, China). Sodium chloride (PubChem CID: 5234; food grade; ≥98%) was purchased from China National Salt Industry Group Co., Ltd. (Beijing, China).

### 2.2 Methods

The experimental methods include the screening and identification of OACs, recovery of condensed water in the extraction stage of tea, salting-out re-distillation to enrich OACs, optimization of recovering conditions, and analytical methods. To obtain the desired results, we designed the recovery processes in detail before experiments. The schematic diagram of the experiments of the recovery processes is shown in Figure S1, which systematically shows the experimental processes and parameter optimization.

#### 2.2.1 Condensed water recovery

The condensed water was recovered according to our previous research with some modifications (Wang, He, Zhang, Du, Xiao, & Xu, 2020). A total of 30 kg Pu-erh tea and 240 L reverses osmosis (RO) water (approximately 5 ℃) were added into a 500 L double-layer extractor equipped with a condensed water recovery vessel (CTQ500L, Pharm-Tech [Tianjin] Co., LTD, Tianjin, China), and soaked for a certain time (0 h, 1 h, 2 h, 3 h, and 4 h) at constant temperature (approximately 5 ℃). Then, steam was introduced into the jacket of extractor for heating (0.20 MPa, about 120 ℃). The released water vapor (rich in OACs) was cooled by tubular cooler with low-temperature recycling water (about 0–5 ℃), and recovered according to the released behavior of OACs of Pu-erh tea. The extraction time was 60 min for three times. The solution of Pu-erh tea was centrifuged, combined, and then concentrated to 40%–45% of solid content.

#### 2.2.2 Further enrichment of odor-active compounds and drying of tea concentrate

Salting-out re-distillation (SRD): To further concentrate the OACs solution, the recovered condensed water and a certain amount of sodium chloride were added into a 100 L bidirectional steel (2205) distiller (Jiangsu Shajiabang Pharmaceutical Chemical Equipment Limited Byshare Ltd.) for SRD. Steam was introduced into the jacket of distiller for heating, and the released water vapor (rich in OACs) was cooled by tubular cooler (about 0–5 ℃) and recovered. To obtain the optimal scheme of SRD, we studied the effects of the amount of sodium chloride (0%, 10%, 20%, 30%, and 40% of condensed water weight), recovery volume of condensed water of re-distillation (2%, 4%, 6%, 8%, and 10% of the volume of condensed water recovered for the first time), and re-use times of sodium chloride (1, 2, 3, 4, 5, and 6 times) systematically.

Membrane treatment: The recovered condensed water was also concentrated using a reverse osmosis membrane (ROM; commonly used in instant tea production) equipment equipped with 1.2 m^2^ membrane element (Dalian Yidong Membrane Engineering Equipment Co., Ltd.). The filtration rate and operating pressure were 24 L/h and 200 Pa, respectively. The volume of OACs aqueous solution was the same as that obtained by salting-out re-distillation.

Drying of tea concentrate: The recovered OACs aqueous solution was added to the concentrate solution of Pu-erh tea and mixed evenly. The drying process and parameters were determined according to our previous research (Wang et al., 2022). Freeze-drying was conducted by spreading solution of Pu-erh tea with a thickness of 0.8–1.0 cm on a stainless steel plate and drying it in a freeze dryer (SQ EL–85, SP Scientific, USA), in which the initial temperature –45 ℃, kept for 240 min, and then programmed from –45 ℃ to 27 ℃ for 2790 min.

#### 2.2.3 Identification and quantitative of odor-active compounds

Headspace solid-phase micro-extraction (HS–SPME): The volatile compounds in Pu-erh tea were extracted by HS–SPME, and the extraction conditions were in accordance with our previous study (Xu et al., 2016). *n*-Decanol (0.1 mL, 6.632 µg/mL) was used as internal standard and added into the sample bottle together with the Pu-erh tea and ultra-pure water.

Liquid–liquid extraction: The OACs in condensed water were extracted by transferring 5 mL of condensed water and 100 μL of internal standard (*n*-decanol, 66.5 μg/mL) into 10 mL glass test tubes (15 mm × 150 mm), and extracting it with 2 mL of *n*-hexane. The extract solvent was dried with anhydrous sodium sulfate overnight before instrumental analysis.

Gas chromatography–olfactometry (GC–O) analysis: The OACs were screening by using Thermo Trace 1300 gas chromatography (Thermo Fisher Scientific Inc., USA) equipped with olfactometry (ODP3, Gerstel, Inc., Germany). A capillary column (RTX-5MS [30 m × 0.25 mm i.d., 0.25 µm film thickness; Restek, Bellefonte, PA]) was used for separation. The effluents were equally divided (1:1) by a “Y” shaped glass splitter at the end of column. The carrier gas rate, injector temperature, and detector temperature were 1 mL/min, 250° C, and 280° C, respectively. Temperature program: initial 60 °C, increased to 180 °C at 2 °C/min; and then increased to 250 °C at 10 °C/min, hold for 3 min. The Humidified air rate and transfer line temperature of olfactometer were 60 mL/min and 250° C, respectively. The six panelists, four males and two females, with ages between 22 and 41 years old were also selected to perform the GC–O analysis. All the assessors were trained for 25 h over a period of 50 d with 10 odor-active standards (*α*-terpineol, *β*-linalool, linalool oxide III, *β*-ionone, dihydro-*β*-ionone, geranyl acetone, 1,2,3-trimethoxybenzene, dihydroactinidiolide, (*E*,*Z*)-2,6-nonadienal, and citral) in Pu-erh tea detected by gas chromatography-mass spectrometry (GC–MS), until every assessor was well familiar with the aroma characteristic of each selected compound. During sniffing, the assessors recorded the perceived aroma, including retention time and aroma descriptor. After GC–O analysis, the perceived OACs were identified by GC–MS.

GC–MS analysis: The OACs were identified by Thermo Trace 1300 GC equipped with ISQ MS. The column, injector temperature, flow rate of carrier gas (helium, >99.999%), and temperature program were consistent with GC–O analysis. The EI energy, mass scan range, interface temperature, ion source temperature, quadrupole temperature, and solvent delay time were 70 eV, 35–450 aum, 280 °C, 230 °C, 150 °C, and 3.0 min, respectively. Identification of OACs was initial compared with the commercial databases (NIST11.L and Wiley7), and further verified by retention indices (*RIs*) and corresponding commercial authentic standards. Quantification of the OACs was performed using internal standard method (*n*-decanol), and the content of each OAC=(the peak area of OAC × the content of internal standard)/the peak area of internal standard.

#### 2.2.4 Sensory analysis of instant Pu-erh tea

Twelve panelists (eight males and four females with ages between 22 and 46 years old) were selected and trained based on GB/T 16291.1–2012 (Sensory analysis–General guidance for instant Pu-erh tea the selection, training, and monitoring of assessors) to evaluate the sensory qualities of IPT samples. Seven scents (stale/musty, woody, floral, fruity, sweet, caramel-like, and off-flavor) and their intensity ranges (1–9: 1-weak, 5-moderate, and 9-extreme) were selected based on our previous study (Du, Wang, Zhang, Ma, Xu, & Xiao, 2019). The intensity ratings were determined by referencing the 1-butanol solutions with different concentrations (Zhu, Niu, & Xiao, 2020). The sensory evaluation was introduced to evaluate the entire aroma coordination (1–9: 1-poor, 5-moderate, and 9-extreme) of IPT samples.

The sensory qualities of IPT samples were evaluated according to the methods of GB/T 31740.1–2015 (Tea products-Part 1: Instant tea in solid form). IPT (0.5 g) was infused with 150 mL of freshly water (85 ± 5 ℃) in a 250 mL transparent glass. The infused samples were coded using three-digit numbers and randomly provided to each panelist. The intensity of each scent of the samples were evaluated and recorded by each panelist. The intensity of each scent was expressed as the average of the score from all panelists. All samples were assessed thrice by each panelist.

#### 2.2.5 Statistical analysis

The data were preprocessed by using MS Excel 2010. Figures were drawn using OriginPro software (version 9.5.1, OriginLab Inc., USA). Duncan’s multiple range tests were performed to test the significance of the differences among different treatments in the investigation of influencing factors using SPSS statistics 17.0 software (SPSS Inc., Chicago, IL, USA).

## 3 Results and discussion

### 3.1.Identification and quantification of odor-active compounds in Pu-erh tea

Forty-four OACs were captured and identified in Pu-erh tea, including 12 alcohols, 8 ketones, 11 aldehydes, 9 methoxy-phenolic compounds, 4 other compounds (Table 1). Among these compounds, the content of 1,2,3-trimethoxybenzene was the highest, at 7.31 mg/kg, followed by linalool oxide Ⅳ (6.50 mg/kg), linalool oxide Ⅱ (5.32 mg/kg), linalool oxide III (4.97 mg/kg), 1,2,4-trimethoxybenzene (4.43 mg/kg), linalool oxide I (4.29 mg/kg), *α*-terpineol (3.56 mg/kg), benzaldehyde (2.82 mg/kg), benzeneacetaldehyde (2.50 mg/kg), dihydroactinidiolide (2.26 mg/kg), 1,2-dimethoxybenzene (1.86 mg/kg), *β*-linalool (1.61 mg/kg), *n*-hexadecanoic acid (0.93 mg/kg), *β*-cyclocitral (0.77 mg/kg), and *β*-ionone (0.70 mg/kg). The results are basically consistent with previous reports (Pang et al., 2019).

**Table 1:** Detection results of the odor-active compounds of Pu-erh tea recovered by different methods.

**Retention time (RT)**	**Retention indices (RI)**	**Reported RI**	**Odor-active compounds**	**Odor descriptors**	**Pu-erh tea (mg/kg)**	**Condensed water of first distillation**		**Condensed water of salting-out re-distillation**		**Condensed water treated with membrane**	
						**Content (mg/L)**	**Recovery rate (%)**	**Content (mg/L)**	**Recovery rate (%)**	**Content (mg/L)**	**Recovery rate (%)**
**Alcohols**					**28.19**	**12.99**	**84.40**	**189.17**	**73.85**	**79.57**	**31.06**
4.35	869	871	1-Hexanol	Fruity	0.30	0.13 ± 0.03	78.72	1.35 ± 0.10	49.17	----	----
7.26	977	981	1-Octen-3-ol	Mushroom	0.44	----	----	----	----	----	----
11.17	1077	1078	Linalool oxide I	Floral, woody	4.29	2.00 ± 0.16	84.03	26.13 ± 0.70	66.93	5.31 ± 1.14	13.60
11.94	1095	1090	Linalool oxide Ⅱ	Floral, woody	5.32	2.59 ± 0.16	89.28	36.92 ± 1.36	76.40	12.01 ± 2.57	24.85
12.51	1107	1105	*β*-Linalool	Floral, fruity	1.61	0.80 ± 0.07	91.74	11.96 ± 1.46	81.96	7.08 ± 2.42	48.50
16.11	1171	1172	Linalool oxide III	Floral, woody	4.97	2.20 ± 0.34	81.17	32.75 ± 4.63	72.51	18.80 ± 2.17	41.62
16.50	1179	1176	Linalool oxide Ⅳ	Floral, woody	6.50	3.03 ± 0.61	85.47	46.67 ± 6.99	79.02	17.53 ± 2.59	29.69
17.40	1195	1191	*α*-Terpineol	Floral, mint	3.56	1.70 ± 0.12	87.72	25.02 ± 1.11	77.41	13.69 ± 2.58	42.35
19.67	1247	1233	(*Z*)-Geraniol	Floral, rose-like	0.12	0.05 ± 0.01	76.41	0.79 ± 0.04	69.97	0.37 ± 0.04	32.71
21.28	1286	1260	Geraniol	Floral, rose-like	0.48	0.24 ± 0.03	93.47	3.80 ± 0.23	87.14	1.76 ± 0.33	40.38
25.86	1362	1336	*β*-Cyclohomogeraniol	Floral	0.55	0.23 ± 0.04	77.51	3.44 ± 0.26	68.62	2.83 ± 1.09	56.47
39.68	1585	1570	Nerolidol	Fruity	0.05	0.02 ± 0.01	90.51	0.34 ± 0.32	81.75	0.19 ± 0.05	45.99
**Ketones**					**2.34**	**1.08**	**84.49**	**15.89**	**74.69**	**7.19**	**33.79**
7.56	987	990	6-Methyl-5-hepten-2-one	Fruity	0.08	0.04 ± 0.01	79.44	0.45 ± 0.09	60.08	0.06 ± 0.03	8.06
11.01	1073	1080	(*E*,*E*)-3,5-Octadien-2-one	Grassy	0.22	0.11 ± 0.04	95.17	1.52 ± 0.10	75.83	0.79 ± 0.03	39.43
13.47	1124	1126	Isophorone	Mint	0.53	0.23 ± 0.03	80.65	3.32 ± 0.39	69.54	0.12 ± 0.04	2.54
31.59	1447	1439	*α*-Ionone	woody	0.08	0.03 ± 0.01	70.00	0.46 ± 0.09	63.33	0.28 ± 0.08	38.33
31.82	1451	1460	Dehydro-*β*-ionone	Woody	0.38	0.19 ± 0.05	88.53	2.88 ± 0.41	82.62	1.48 ± 0.27	42.40
32.23	1456	1438	Dihydro-*β*-ionone	Woody	0.02	0.01 ± 0.002	67.27	0.10 ± 0.04	60.00	0.06 ± 0.04	36.36
33.25	1471	1449	Geranyl acetone	Floral, sweet	0.33	0.15 ± 0.04	85.48	2.28 ± 0.21	76.34	1.83 ± 0.34	61.32
35.04	1497	1494	*β*-Ionone	Woody, floral	0.70	0.32 ± 0.07	84.04	4.88 ± 0.38	76.48	2.57 ± 1.17	40.29
**Aldehydes**					**8.77**	**4.03**	**84.09**	**52.95**	**66.44**	**13.19**	**16.55**
4.11	853	858	(*E*)-2-Hexenal	Green, leaf-like	0.44	0.23 ± 0.04	95.20	2.55 ± 0.30	63.12	----	----
4.97	905	904	Heptanal	Pungent	0.48	0.18 ± 0.04	67.56	1.87 ± 0.22	42.49	----	----
6.67	959	963	Benzaldehyde	Almond	2.82	1.33 ± 0.15	86.21	17.21 ± 1.14	67.17	2.61 ± 0.47	10.18
8.45	1011	1006	(*E*,*E*)-2,4-Heptadienal	Green, fatty	0.39	0.14 ± 0.04	65.79	1.79 ± 0.36	50.30	0.44 ± 0.12	12.34
9.83	1044	1049	Benzeneacetaldehyde	Green, sweet	2.50	1.18 ± 0.15	86.95	15.51 ± 2.01	68.29	1.86 ± 0.14	8.19
12.75	1111	1105	Nonanal	Fatty, fruity	0.37	0.15 ± 0.04	73.00	2.00 ± 0.24	60.00	0.52 ± 0.21	15.64
15.34	1158	1155	(*E*,*Z*)-2,6-Nonadienal	Green, cucumber-like	0.21	0.07 ± 0.02	61.36	0.98 ± 0.17	52.44	0.28 ± 0.02	14.94
15.70	1164	1161	(*E*)-2-Nonenal	Green, cucumber-like	0.21	0.10 ± 0.03	91.72	1.49 ± 0.27	79.87	0.17 ± 0.03	9.09
17.91	1205	1191	Safranal	Herbal, woody	0.23	0.12 ± 0.04	96.22	1.78 ± 0.06	85.32	0.82 ± 0.17	39.39
19.14	1235	1227	*β*-Cyclocitral	Fruity, sweet	0.77	0.37 ± 0.08	87.34	5.53 ± 0.91	78.87	4.73 ± 1.39	67.46
22.22	1305	NF	Citral	Lemon-like	0.35	0.16 ± 0.05	81.29	2.24 ± 0.26	70.18	1.76 ± 0.29	55.18
**Methoxy-phenolic compounds**					**14.99**	**6.85**	**83.90**	**104.86**	**77.03**	**62.08**	**45.58**
15.02	1152	1144	1,2-Dimethoxybenzene	Stale	1.86	0.86 ± 0.08	84.75	12.68 ± 0.86	74.81	4.01 ± 1.35	23.65
15.90	1167	1163	1,4-Dimethoxybenzene	Stale	0.31	0.16 ± 0.04	98.03	2.24 ± 0.31	80.77	0.48 ± 0.11	17.27
20.38	1264	1246	3,4-Dimethoxytoluene	Stale	0.31	0.11 ± 0.03	66.67	1.66 ± 0.22	59.50	1.14 ± 0.31	40.83
24.89	1347	1312	1,2,3-Trimethoxybenzene	Stale	7.31	3.40 ± 0.49	85.32	52.83 ± 5.42	79.55	35.29 ± 6.97	53.13
25.50	1357	1323	4-Ethyl-1,2-dimethoxybenzene	Stale	0.23	0.12 ± 0.04	94.32	1.70 ± 0.20	81.66	1.09 ± 0.43	52.40
26.69	1375	1369	1,2,4-Trimethoxybenzene	Stale	4.43	2.07 ± 0.35	85.76	31.95 ± 4.15	79.34	18.81 ± 4.92	46.71
28.10	1397	1367	4-Ethenyl-1,2-dimethoxybenzene	Stale	0.11	0.04 ± 0.01	61.76	0.54 ± 0.04	55.80	0.30 ± 0.07	31.03
32.94	1467	1454	1,2,3,4-Tetramethoxybenzene	Stale, musty	0.19	0.09 ± 0.04	87.72	1.26 ± 0.36	72.98	0.93 ± 0.52	53.86
35.81	1510	NF	Methyleugenol	Anise-like	0.24	----	----	----	----	----	----
**Other compounds**					**3.50**	**1.52**	**79.69**	**19.37**	**60.76**	**13.72**	**43.04**
36.77	1529	1519	2,4-Di-*tert*-butylphenol	Green, herbal	0.25	0.11 ± 0.04	77.50	1.42 ± 0.06	61.71	1.02 ± 0.15	44.34
37.22	1538	1537	Dihydroactinidiolide	Woody, coumarin	2.26	1.06 ± 0.32	86.20	14.04 ± 4.62	68.22	10.29 ± 6.09	50.01
39.72	1586	1588	Geranyl isovalerate	Fruity	0.06	----	----	----	----	----	----
60.63	1970	1978	*n*-Hexadecanoic acid	Rancid, pungent	0.93	0.35 ± 0.07	69.43	3.91 ± 0.83	46.17	2.41 ± 1.77	28.45
**Total**					**57.79**	**26.47**	**83.94**	**382.24**	**72.79**	**175.75**	**33.46**

“----”: Not detected; “NF”: Not found.

The reported RIs were queried from NIST Chemistry WebBook (https://webbook.nist.gov/chemistry/).

### 3.2 Optimization of recovering conditions

Based on the screening and identification of OACs, the influencing factors related to the recovery of OACs of Pu-erh tea were optimized to determine the most effective recovery conditions. The optimization results are as follows.

#### 3.2.1 Influence of soaking time on the releasing rate of odor-active compounds

**Fig. 1:**
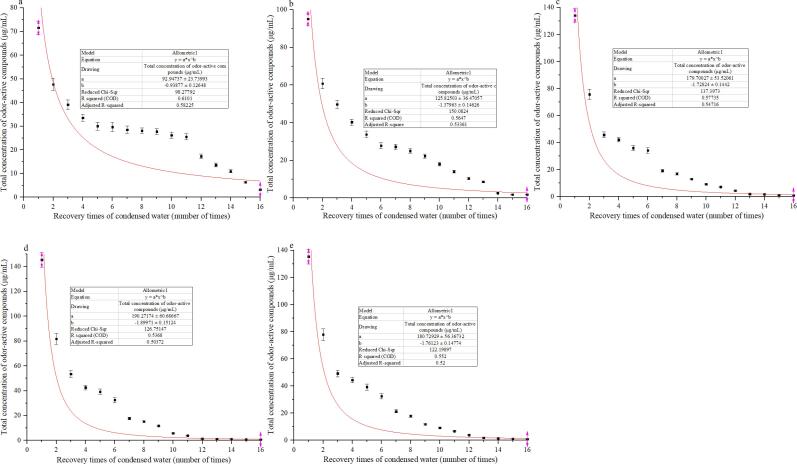
Effect of soaking time of Pu-erh tea on releasing rate of odor-active compounds (a-0 h, b-1 h, c-2 h, d-3 h, e-4 h; 3 replications).

After soaking, the OACs are easily to release from Pu-erh tea (Du, Wang, Li, Xiao, Li, & Xu, 2013). To evaluate the effect of soaking time on the releasing rate of OACs, we continuously recovered the condensed water during the water-boiling extraction of Pu-erh tea, with a volume of 5000 mL each time until the sensory flavor was very light, and then non-linear curve fitting analysis was performed using the recovery times (*x*) and the corresponding total concentrations (*y*) of OACs as described by de Lemos Sampaio, Biasoto, Marques, Batista, & Silva (2013). The results showed that all the data could be significantly fitted (*p* less than 0.01) to a power function type curve (Fig. 1 a–e). The exponential coefficients of the fitted models showed that soaking for a certain time could accelerate the release of OACs. With prolonged soaking time, the release rate of OACs gradually increased and reached the maximum when the soaking time was 3 h. Under this soaking condition, the OACs have been basically completely released after recovered 11 times, that is, 55 L condensed water has been recovered. Therefore, the volume of the condensed water recovered for the first time is 55 L.

#### 3.2.2 Influence of amount of sodium chloride on odor-active compounds recovery

**Fig. 2:**
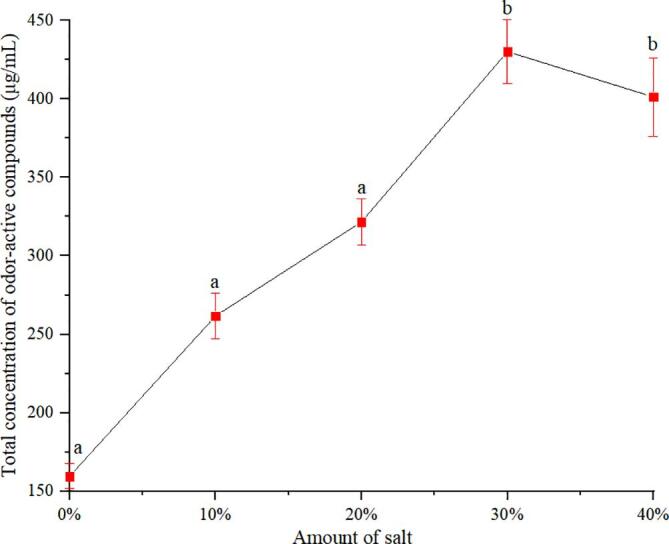
Effect of the amount of sodium chloride on the recovery efficiencies of odor-active compounds (the error bars represent standard deviation [SD] of 3 replications; different letters indicate significant differences [*p* less than 0.05, ANOVA, Duncan’s multiple range test]).

The addition of inorganic salts to aqueous solution caused the abrupt weakening of the solvation forces between the water molecules and target compounds and increased the release rate of target compounds during distillation (Tsai et al., 2017, Wang et al., 2012). To screen the most suitable amount of sodium chloride for OACs recovery, we investigated different amounts of sodium chloride, as illustrated in Fig. 2. The recovery yield of total OACs increased with the increase in salt amount, because the addition of some inorganic salt to the condensed water could reduce the solubility of OACs, and the OACs were easily released. The optimum recovery yield was reached when the salt addition amount was 30%. With the increasing salt amount, the recovery yield of OACs did not increase significantly. Considering the effect of the increase of salt amount on the equipment and the difficulty of salt recovery, 30% salt addition was selected.

#### 3.2.3 Recovery volume of condensed water of SRD

**Fig. 3:**
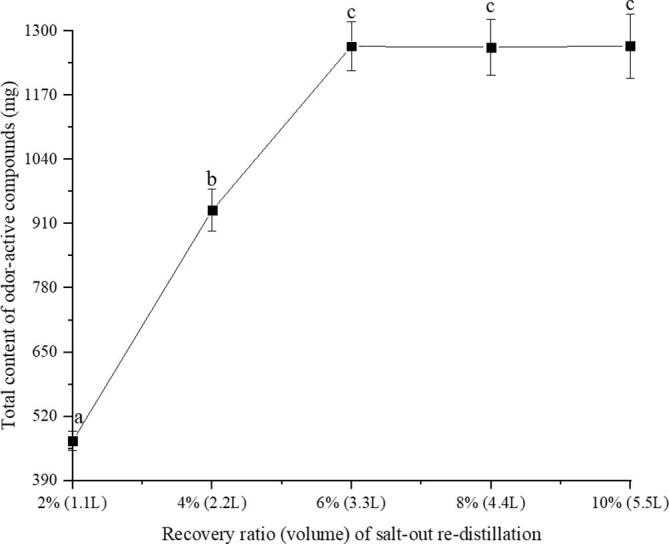
Effect of the recovery volume of condensed water of re-distillation on the recovery efficiencies of odor-active compounds (the error bars represent SD of 3 replications; different letters indicate significant differences [*p* less than 0.05, ANOVA, Duncan’s multiple range test]).

To ensure the effective recovery of OACs, the recovery volume of condensed water of SRD was investigated, as illustrated in Fig. 3. The total content of OACs increased with the increasing in the recovered volume of condensed water, and reached the optimum value when the recovered volume was 6% of the first recovered condensed water. By increasing of recovered volume of condensed water, the recovery yield of OACs did not increase significantly. Considering that the condensed water needs to be added to the concentrated juice of tea for freeze-drying, 6% (3.3 L) of the first recovered condensed water was recovered.

#### 3.2.4 Influence of re-use times of sodium chloride on odor-active compounds recovery

**Fig. 4:**
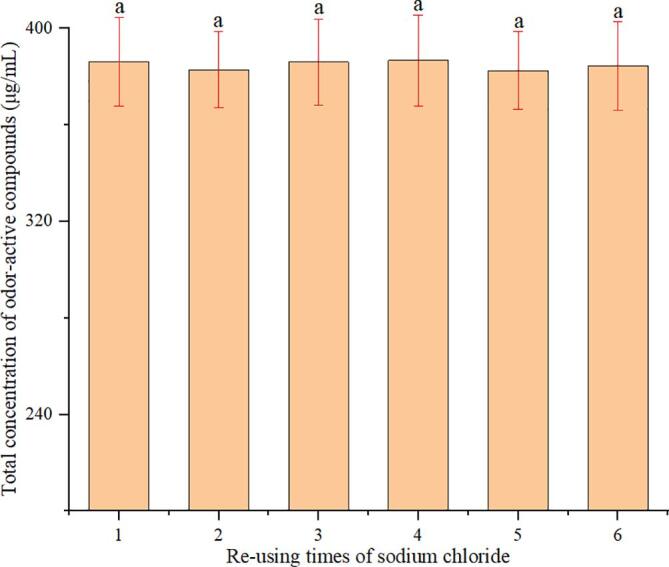
Effect of the re-use times of sodium chloride on the recovery efficiencies of odor-active compounds (the error bars represent SD of 3 replications; different letters indicate significant differences [*p* less than 0.05, ANOVA, Duncan’s multiple range test]).

The re-use of sodium chloride can effectively reduce costs and achieve cleaner production. To achieve greener and pollution-free production, the influence of re-use times of sodium chloride was evaluated. After each salting-out re-distillation, sodium chloride was recovered by evaporation and used again, and no significant differences (*p* > 0.05) were observed in the total concentrations of OACs when the sodium chloride re-use for six times (Fig. 4). The results showed that the re-use of sodium chloride slightly affected the recovery of OACs of Pu-erh tea, and it could achieve the re-use of sodium chloride.

### 3.5 Recovery of odor-active compounds from Pu-erh tea

According to the released behavior of OACs, 41 OACs were recovered in the first distillation, and the total recovery rate of OACs was 83.94% (Table 1). And then, the OACs were further enriched using salting-out re-distillation (SRD) and reverse osmosis membrane (ROM). Forty-one and thirty-eight OACs were recovered via SRD and ROM, respectively (Table 1). The total recovery rate of OACs reached 72.29% using SRD, among them, the recovery rate of methoxy-phenolic compounds was the highest, at 77.03%, followed by ketones (74.69%), alcohols (73.85%), and aldehydes (66.44%). Compared with SRD, the total recovery rate of OACs is relatively low using ROM, only 33.46%, among them, the recovery rates of methoxy-phenolic compounds, ketones, alcohols, and aldehydes were less than 50%. The recovery rates of total and each kind of OACs using SRD were significantly higher than that using ROM. Meanwhile, the recovery efficiency of SDR is significantly higher than that of oil adsorption method (39.53%) (Wang, He, Zhang, Du, Xiao, & Xu, 2020).

#### 3.5.1 Alcohols

Eleven and ten alcohol compounds were recovered via SRD and ROM, respectively. Using SRD, the recovery rate of geraniol was the highest, at 87.14%, followed by *β*-linalool (81.96%), nerolidol (81.75%), linalool oxide Ⅳ (79.02%), *α*-terpineol (77.41%), linalool oxide Ⅱ (76.40%), linalool oxide III (72.51%), (Z)-geraniol (69.97%), *β*-cyclohomogeraniol (68.62%), linalool oxide I (66.93%), and 1-hexanol (49.17%). While with ROM, the recovery rates of these alcohol compounds ranged from 0 to 56.47%, among which the recovery rate of *β*-cyclohomogeraniol was the highest, and the recovery rates of other compounds were less than 50% (Table 1).

Among these compounds, geraniol, (Z)-geraniol, and nerolidol were described as smelling like ‘rose’ and ‘floral’ odors in Pu-erh tea. Geraniol is an extremely potent aroma compound with a threshold of 10 μg/kg, which can exhibit high odor intensity even at low concentration (Du, Plotto, Baldwin, & Rouseff, 2011). As a potential sesquiterpene aroma compound, nerolidol was detected in many teas, especially in Oolong tea at high concentration, which was considered to be one of the key OACs for the high quality Oolong tea (Ma, Qu, Zhang, Qiu, Wang, & Chen, 2014). These compounds were considered to be the main contributors to the floral fragrance of Pu-erh tea. *β*-Linalool was described as smelling like ‘floral’ and ‘fruity’ odors in Pu-erh tea, which underwent significant oxygenation during the post-fermented process, and its content was lower in ripened Pu-erh tea than in raw Pu-erh tea (Xu et al., 2016). Linalool oxides (Ⅰ, Ⅱ, III and Ⅳ) were identified to provide ‘floral’ and ‘woody’ odors in Pu-erh tea, which were detected with higher concentration in semi fermented, fermented, and post-fermented teas than in their raw materials (Feng et al., 2019, Pang et al., 2019). *α*-Terpineol was described as smelling like ‘floral’ and ‘mint’ fragrances in Pu-erh tea, which was considered to be the main contributor to the formation of the unique aroma of Pu-erh tea (Lv et al., 2012). *β*-Cyclohomogeraniol was identified to provide ‘floral’ odor in Pu-erh tea, which was detected as one of the main OACs in the condensed water of Pu-erh tea (Wang, He, Zhang, Du, Xiao, & Xu, 2020). 1-Hexanol was described as smelling like ‘fruity’ fragrance, which was detected to present a low odor intensity in Pu-erh tea (Xu et al., 2016). Most of these alcohols are unsaturated alcohols with low odor thresholds values and have strong odor intensities. Effective recovery these alcohol compounds can significantly improve the flavor quality of final products of Pu-erh tea.

#### 3.5.2 Ketones

Eight ketone compounds were recovered via both SRD and ROM. Using SRD, the recovery rate of dehydro-*β*-ionone was the highest, at 82.62%, followed by *β*-ionone (76.48%), geranyl acetone (76.34%), (*E*,*E*)-3,5-octadien-2-one (75.83%), isophorone (69.54%), *α*-ionone (63.33%), 6-methyl-5-hepten-2-one (60.08%), and dihydro-*β*-ionone (60.00%). While with ROM, the recovery rates of these ketones ranged from 2.54% to 61.32%, among which the recovery rate of geranyl acetone was the highest, and the recovery rates of other compounds were less than 50%.

*α*-Ionone, *β*-ionone, dehydro-*β*-ionone, and dihydro-*β*-ionone, which comprise a group of extremely potent volatile compounds with low odor thresholds, especially dihydro-*β*-ionone and *β*-ionone with the values of 0.001 µg/kg and 0.007 µg/kg, respectively (Xu et al., 2019). These compounds were considered to be the main contributors to the ‘woody’ fragrance of Pu-erh tea (Lv et al., 2015). Geranyl acetone was described as smelling like ‘rose’ and ‘green’ odors in Pu-erh tea, which was considered to be the main contributor to the formation of the characteristic aroma of Pu-erh tea (Lv et al., 2012). (*E*,*E*)-3,5-Octadien-2-one was described as smelling like ‘grass’ odor, which was detected as the primary aroma compound degraded during the post-fermentation of Pu-erh tea (Ma et al., 2021). Isophorone and 6-methyl-5-hepten-2-one were described as smelling like ‘mint’ and ‘fruity’ odor, respectively, which were identified as the major compounds to the aroma profile of Pu-erh tea (Wang, He, Zhang, Du, Xiao, & Xu, 2020).

#### 3.5.3 Aldehydes

Eleven and nine aldehyde compounds were recovered via SRD and ROM, respectively. Using SRD, the recovery rate of safranal was the highest, at 85.32%, followed by (*E*)-2-nonenal (79.87%), *β*-cyclocitral (78.87%), citral (70.18%), benzeneacetaldehyde (68.29%), benzaldehyde (67.17%), (*E*)-2-hexenal (63.12%), nonanal (60.00%), (*E*,*Z*)-2,6-nonadienal (52.44%), (*E*,*E*)-2,4-heptadienal (50.30%), and heptanal (42.49%). While with ROM, the recovery rates of these ketones ranged from 0 to 67.46%, among which the recovery rate of *β*-cyclocitral was the highest, followed by citral (55.18%), and the recovery rates of other compounds were less than 50%.

Most of these aldehydes originated from thermal Strecker oxidative degradation of amino acids and fatty acids (Barra, Baldovini, Loiseau, Albino, Lesecq, & Lizzani Cuvelier, 2007), which considered to be important for the entire aroma of Pu-erh tea because of their relatively low odor threshold values (Selli & Cayhan, 2009). Safranal was described as smelling like ‘herbal’ and ‘woody’ fragrances, which was detected to present a low dilution factor (FD) factor in Pu-erh tea (Zhang et al., 2019). (*E*)-2-Nonenal and (*E*,*Z*)-2,6-nonadienal were identified to provide ‘green’ and ‘cucumber-like’ odors. (*E*)-2-Nonenal was detected in Pu-erh tea with a high FD factor, and (*E*,*Z*)-2,6-nonadienal was considered to be extremely potent aroma compounds in Pu-erh tea due to its relatively low threshold (0.2 μg/L) (Pang et al., 2019). *β*-Cyclocitral wad identified to present ‘fruity’ and ‘sweet’ odors, which was detected in IPT tea with a medium odor intensity (Wang et al., 2022). Citral was described as smelling like ‘lemon’ odor, which was detected to have a relatively low content in Pu-erh tea. Benzeneacetaldehyde was identified to provide ‘green’ and ‘sweet’ fragrances, which was detected in IPT with a high FD factor (Zhang et al., 2019). Benzaldehyde was described as smelling like ‘almond’ odor in Pu-erh tea. As an crucial odor-active compound, benzaldehyde exists in many kinds of teas. (*E*)-2-Hexenal was described as smelling like ‘green’ and ‘leaf’ fragrances, which was identified as one odor-active compound in the condensed water of Pu-erh tea (Wang, He, Zhang, Du, Xiao, & Xu, 2020). Nonanal was identified to provide ‘fatty’ and ‘fruity’ odors, which was determined to play a decisive role in the formation of the entire aroma of Pu-erh tea (Pang et al., 2019). (*E*,*E*)-2,4-Heptadienal was identified to provide ‘green’ and ‘fatty’ odors, which was identified as a key odor-active compound in Pu-erh tea (Du, Li, Li, Li, Li, & Xiao, 2014). (*E*)-2-Hexenal was described as smelling like ‘pungent’ odor, which was identified as one odor-active compound in the condensed water of Pu-erh tea (Wang, He, Zhang, Du, Xiao, & Xu, 2020).

#### 3.5.4 Methoxy-phenolic compounds

Eight methoxy-phenolic compounds were recovered via both SRD and ROM. Using SRD, the recovery rate of 4-ethyl-1,2-dimethoxybenzene was the highest, at 81.66%, followed by 1,4-dimethoxybenzene (80.77%), 1,2,3-trimethoxybenzene (79.55%), 1,2,4-trimethoxybenzene (79.34%), 1,2-dimethoxybenzene (74.81%), 1,2,3,4-tetramethoxybenzene (72.98%), 3,4-dimethoxytoluene (59.50%), and 4-ethenyl-1,2-dimethoxybenzene (55.80%). While with ROM, the recovery rates of these methoxy-phenolic compounds ranged from 17.27% to 53.86%, among which the recovery rate of 1,2,3,4-tetramethoxybenzene was the highest, followed by 1,2,3-trimethoxybenzene (53.13%) and 4-ethyl-1,2-dimethoxybenzene (52.40%), and the recovery rates of other compounds were less than 50%.

Most methoxy-phenolic compounds were identified to provide ‘stale’ and ‘musty’ odors, which were considered to be the most key aroma compounds in the formation of the unique stale flavor of Pu-erh tea. These compounds were believed to directly determine the quality and market value of Pu-erh tea (Lv, Zhong, Lin, Wang, Tan, & Guo, 2012). Methoxy-phenolic compounds was identified as a class of compounds with a relatively high content in Pu-erh tea, which were considered to be formed by the microbial degradation and methylation of tea catechins as well as thermal degradation during the pile-fermentation (Du et al., 2014, Pang et al., 2019, Xu et al., 2005).

#### 3.5.8 Other compounds

Three other compounds were recovered via both SRD and ROM. Using SRD, the recovery rate of dihydroactinidiolide was the highest, at 68.22%, followed by 2,4-di-*tert*-butylphenol (61.71%) and *n*-hexadecanoic acid (46.17%). While with ROM, the recovery rate of dihydroactinidiolide was the highest, at 50.01%, and the recovery rates of 2,4-di-*tert*-butylphenol and *n*-hexadecanoic acid were less than 50%. Dihydroactinidiolide was described as smelling like ‘woody’ odor, which was identified as derived from the degradation of *β*-carotene based on microbial activity or thermal degradation during fermentation processing of Pu-erh tea (Lv et al., 2015). 2,4-Ditert-butylphenol was described as like ‘phenolic’ and ‘herbal’ odors, which was identified as an odor-active compound in Pu-erh and other Dark teas (Ma et al., 2021). *n*-Hexadecanoic acid was described as smelling like ‘rancid’ and ‘pungent’ odors, which was identified as an odor-active compound in Pu-erh tea (Xu et al., 2016).

### 3.6 Sensory evaluation of instant Pu-erh tea

**Fig. 5:**
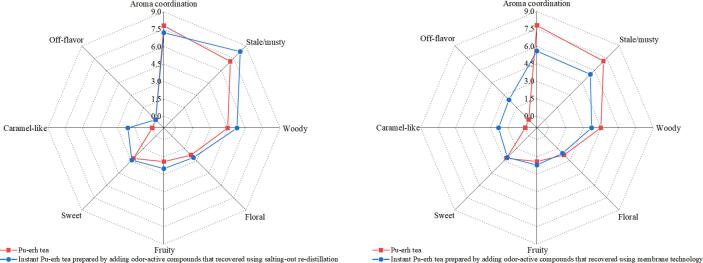
Effect of the recovery methods of odor-active compounds on the sensory quality of instant Pu-erh teas.

The influences of OACs recovery methods on the sensory qualities of IPTs were evaluated. The sensory results of aroma attributes and coordination are shown in Fig. 5. The IPT prepared by adding OACs recovered via SRD demonstrated strong intensities of stale/musty, woody, floral, and fruity attributes than that in Pu-erh tea. This phenomenon may be caused by the different release rates of odor-active compounds in IPT and Pu-erh tea. The caramel-like descriptor of IPT samples could be attributed to the caramelization reaction during high-temperature processing. In terms of aroma coordination, the IPT prepared by adding OACs recovered by SRD was closer to Pu-erh tea. The aroma coordination of IPTs prepared by adding OACs recovered by ROM was relatively poor. This property is quite different from that of Pu-erh tea, possibly because of the loss of most OACs from Pu-erh tea, resulting in the poor entire aroma coordination of IPTs. The sensory evaluation results verified that the effective recovery of OACs lost in the processing of instant could not only improve the intensities of attributes, but also improve the coordination of the entire aroma of IPTs. The superiority of the developed method was proved again.

## 4 Conclusion

Most of OACs that contribute to the aroma profile of tea are lost during the manufacturing process of instant tea or tea beverages, which seriously impacts the sensory quality of final products. In the present study, the method of SDR combined with sensory-directed analysis was developed to recover the released OACs during the processing of IPT. Firstly, the soaking time of tea and recovery volume of condensed water of first distillation were determined based on the released behavior of OACs. Secondly, the effects of amount of sodium chloride, recovery volume of condensed water of re-distillation, and re-use times of sodium chloride on the recovery efficiencies of OACs were studied systematically. Under the optimized conditions, 41 OACs were recovered in the first distillation, and the total recovery rate was 83.94%. Forty-one OACs were recovered via SRD concentration, and the total recovery rate reached 72.29%. While 38 OACs were recovered via ROM concentration, and the recovery rate was only 33.46%. The IPT prepared by adding OACs recovered via SRD demonstrated strong aroma attributes intensities and good aroma coordination. This low-costs, high-recovery-efficiency, and environment-friendly method of recovering OACs from the production process of IPT can provide a more effective scheme to improve the flavor quality of IPT. However, although a relatively good recovery efficiency of Pu-erh tea’s OACs has been obtained in this study, the productive application of this technology needs to deeply optimize the influencing factors and further verify the recovery process.

## Declaration of Competing Interest

The authors declare that they have no known competing financial interests or personal relationships that could have appeared to influence the work reported in this paper.
