# Supplementary material for: Salting-out re-distillation combined with sensory-directed analysis to recover odor-active compounds for improving the flavor quality of instant Pu-erh tea
Source: Food Chem X. 2022 Apr 18;14:100310. doi: 10.1016/j.fochx.2022.100310 (PMC9043642; doi:10.1016/j.fochx.2022.100310)
Supplement: Supplementary data 1 [file mmc1.doc]

**Supplementary material**

Table S1 The information of Pu-erh tea samples

| Sample | Picked and processed time | Source | Sensory evaluation | Manufacturer |
| --- | --- | --- | --- | --- |
| Pu-erh1 | 2014 | Jinggu, Yunan, China | Strong stale and woody odors | Yunnan Tasly Deepure Biological Tea Group Co., Ltd |
| Pu-erh2 | 2014 | Jingdong, Yunan, China | Strong stale and woody odors, faint herbal odor |
| Pu-erh3 | 2015 | Simao, Yunan, China | Strong stale and woody odors, faint herbal odor |
| Pu-erh4 | 2015 | Simao, Yunan, China | Strong stale and woody odors |
| Pu-erh5 | 2017 | Zhenyuan, Yunan, China | Strong stale and woody odors |


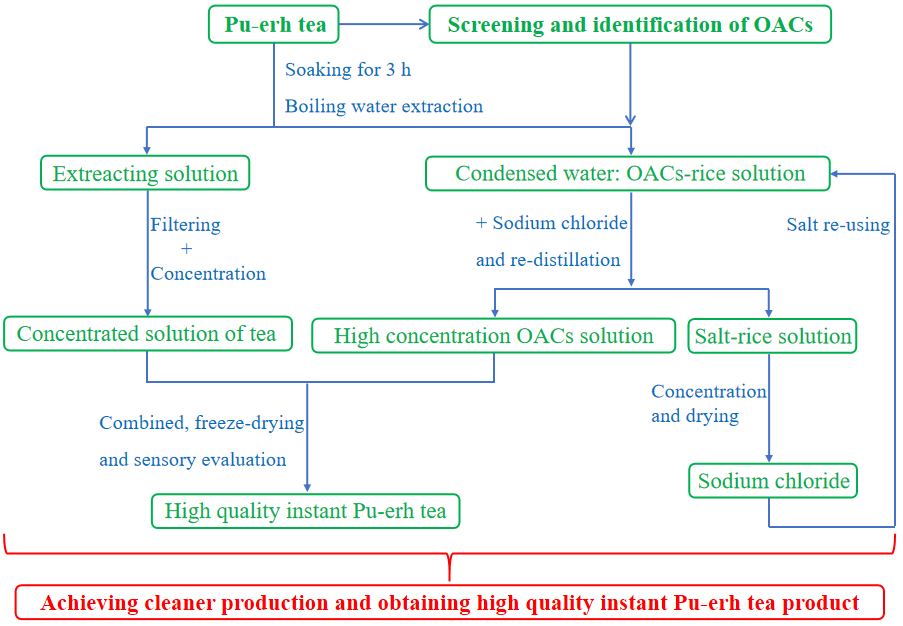


Figure S1 Flowsheet of stepwise odor-active compounds (OACs) recovering during the processing of instant Pu-erh tea
